# Tumor Microenvironment-Specific Chemical Internalization for Enhanced Gene Therapy of Metastatic Breast Cancer

**DOI:** 10.34133/2021/9760398

**Published:** 2021-06-18

**Authors:** Yun Zhou, Mian Yu, Changjun Tie, Yang Deng, Junqing Wang, Yunfei Yi, Fan Zhang, Chenyi Huang, Hairong Zheng, Lin Mei, Meiying Wu

**Affiliations:** ^1^ School of Pharmaceutical Sciences (Shenzhen), Sun Yat-sen University, Shenzhen 518107, China; ^2^ Paul C. Lauterbur Research Center for Biomedical Imaging, Institute of Biomedical and Health Engineering, Shenzhen Institutes of Advanced Technology, Chinese Academy of Sciences, Shenzhen 518055, China; ^3^ Tianjin Key Laboratory of Biomedical Materials, Key Laboratory of Biomaterials and Nanotechnology for Cancer Immunotherapy, Institute of Biomedical Engineering, Chinese Academy of Medical Sciences & Peking Union Medical College, Tianjin 300192, China

## Abstract

Benefiting from treating diseases at the genetic level, gene therapy has been considered a new revolution in the biomedical field. However, the extracellular and intracellular barriers during gene transport such as enzymatic degradation and endo-/lysosomal sequestration significantly compromise the therapeutic efficacy. Though photochemical internalization (PCI) has emerged as a promising approach for causing endo-/lysosomal leakage with translocation of the internalized molecules into the cytosol, its effect is still unsatisfactory due to the insufficient light penetration depth. Here, we develop tumor microenvironment-specific enhanced gene delivery by means of ROS generated from the in situ cascaded catalytic reactions in tumors involving GOx-mediated redox reaction and Mn^2+^-mediated Fenton-like reaction. The efficient enzymatic protection and successful endo-/lysosomal escape of cargo gene complexes have been demonstrated. Moreover, anti-Twist siRNA-loaded G@MMSNs-P exhibit tumor-specific biodegradation, excellent T_1_-weighted MR imaging, and significant inhibitory effects against breast cancer growth and pulmonary metastasis.

## 1. Introduction

Gene therapy has emerged as an attractive technique for treating various genetic and acquired disorders [1–3], which is implemented by transporting therapeutic nucleic acids into cells to correct or modify a specific genetic target responsible for the manifestation of a disease [4]. The key challenge of this technique is to exploit safe and effective gene delivery vectors to protect genetic cargos and facilitate their transfer to the site of action [5–7]. To date, numerous nonviral vectors have been substantially advanced to overcome the extracellular and intracellular barriers during gene transport [8, 9], such as enzymatic degradation [10], cellular internalization [11], and endo-/lysosomal sequestration [12]. However, the gene transfection efficiencies of these vectors are still not satisfactory, thus hindering their further application.

Photochemical internalization (PCI), a spatiotemporally controllable technology [13], has been developed for enhancing cytosolic release of trapped molecules in endocytic vesicles by photochemical disruption of the endo-/lysosomal membrane using light and photosensitizers [14–16]. Light-induced reactive oxygen species (ROS) formation is the critical factor that causes membrane leakage with translocation of the internalized molecules into the cytosol [17, 18]. Although PCI has exhibited significant improvement in gene transfection efficiency, its clinical application is limited by the insufficient light penetration depth. Based on the crucial role of ROS in regulating the biological functions of living organisms, ROS-related biomedical applications have been extensively advanced over the past few decades [19, 20]. Chemodynamic therapy (CDT) is a newly developed tumor therapeutic modality that could convert intracellular hydrogen peroxide (H_2_O_2_) into highly toxic ROS through the Fenton or Fenton-like reaction [21–24]. The imbalance of the intracellular redox/oxidation state could induce severe oxidative damage to organelles and cause remarkable cell apoptosis and necrosis [25–27]. The H_2_O_2_ level in tumor cells is too low to achieve satisfactory therapeutic performance; thus, H_2_O_2_-evolving nanoplatforms are highly needed to enhance the CDT efficacy. Glucose oxidase (GOx), an enzyme catalyzing the oxidization of glucose into gluconic acid and H_2_O_2_, has been extensively used as an “in situ amplifier” to elevate the acidic and H_2_O_2_ level in the tumor microenvironment (TME) [28–30].

**Figure 1:**
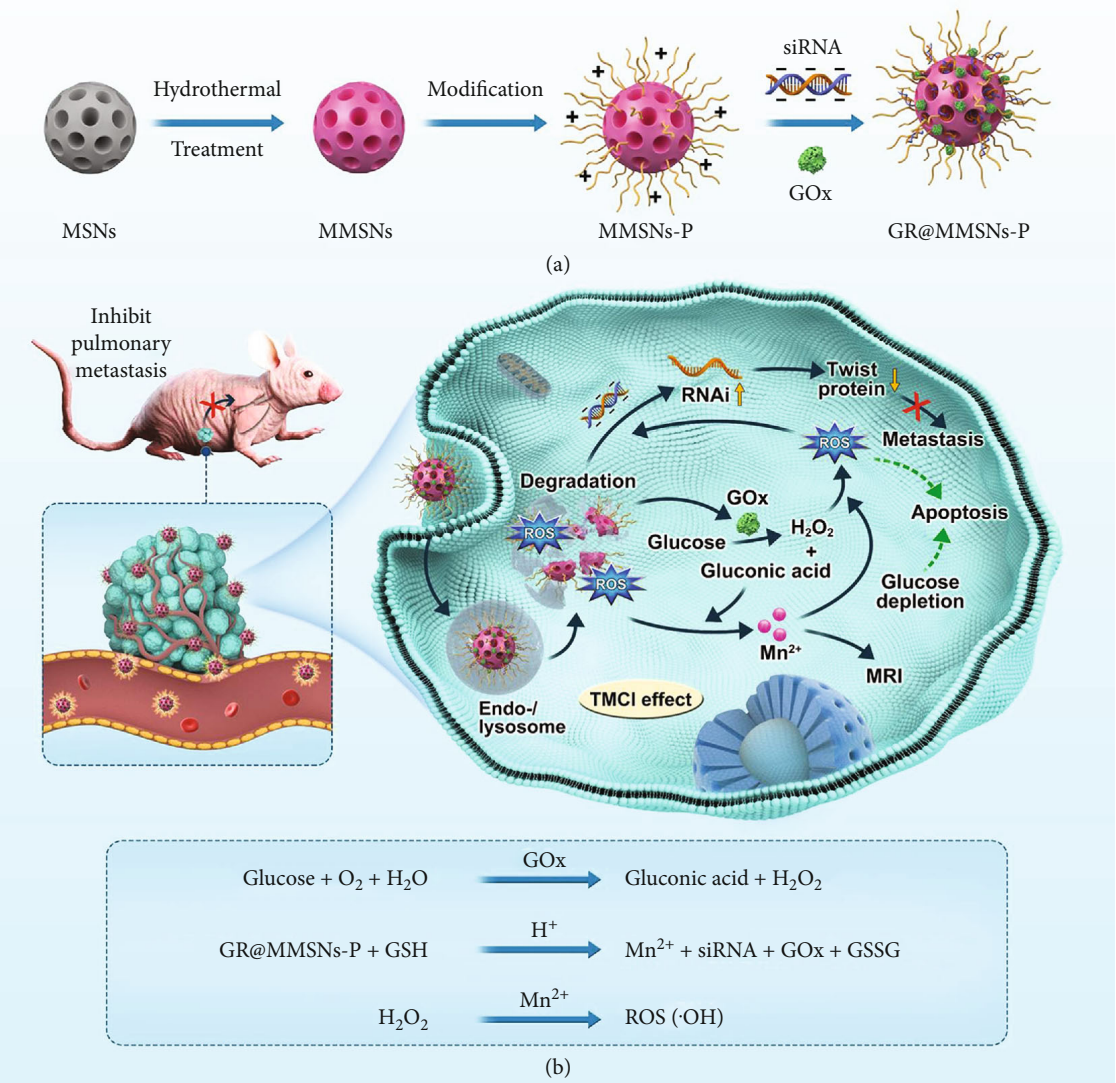
Schematic illustration of the TMCI strategy. (a) Fabrication of GR@MMSNs-P. (b) TME-specific enhanced gene delivery by means of ROS generated from the in situ cascaded catalytic reactions in tumors involving GOx-mediated redox reaction and Mn^2+^-mediated Fenton-like reaction.

Herein, we propose a tumor microenvironment-specific chemical internalization (TMCI) strategy in which ROS can be greatly facilitated to produce GOx-involved Fenton-like reaction, independent of exogenous laser irradiation, achieving destroyed endocytic membrane, promoted endo-/lysosomal escape, and improved gene transfection efficiency. This strategy can be realized by constructing polyethyleneimine- (PEI-) modified mesoporous manganese silicate nanoparticles and subsequently encapsulating GOx and siRNA on the nanoparticle surfaces and/or in the mesoporous channels (designated as GR@MMSNs-P, Figure 1(a)). Antimetastasis siRNA targeting Twist was chosen as the therapeutic nucleic acid due to the essential role of Twist in promoting epithelial-to-mesenchymal transition (EMT) and contributing to tumor metastasis [31–34]. Suppression of Twist expression by RNA interference has been confirmed to be effective in combating tumor metastasis [35, 36].

The fabricated GR@MMSNs-P exhibit the following distinctive superiorities in enhanced gene therapy for metastatic breast cancer (Figure 1(b)). First, Mn-doped MMSNs are prone to collapse once entrapped into the intracellular acidic or reducing microenvironment of tumor cells, which could be accelerated by GOx-mediated gluconic acid production, ensuring the high biocompatibility and biodegradability of nonviral vectors as well as enhanced T_1_-weighted magnetic resonance (MR) imaging. Second, the molecular weight of PEI employed is very low (Mw=5000), endowing MMSNs-P with low toxicity and enhanced cellular uptake efficiency. Last but not least, the intracellular ROS level could be substantially elevated by the in situ cascaded catalytic reactions involving GOx-mediated redox reaction and Mn^2+^-mediated Fenton-like reaction, thus resulting in improved gene transfection efficiency as well as significant inhibition on tumor growth and pulmonary metastasis.

## 2. Results

### 2.1. Fabrication and Characterization of MMSNs

**Figure 2:**
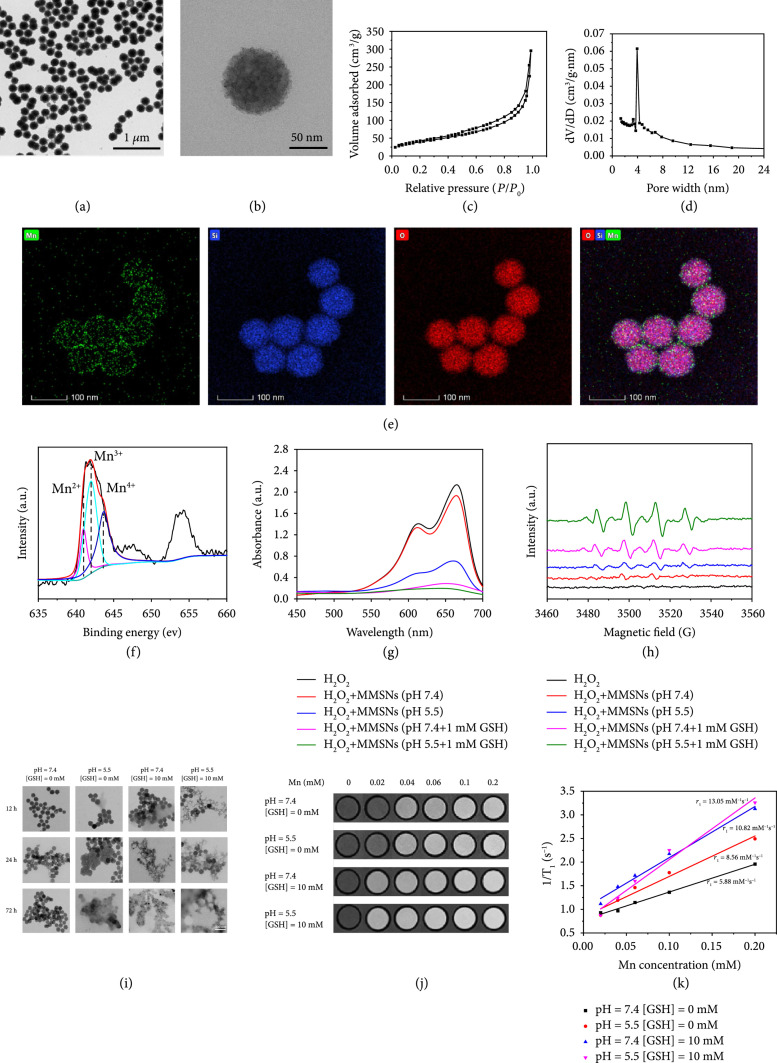
Physicochemical characterization of MMSNs. (a, b) TEM image, (c) N_2_ absorption-desorption isotherm, (d) corresponding pore size distribution, and (e) element mapping of MMSNs. (f) The valence state of Mn in MMSNs. (g) UV-vis absorption spectra of MB after different treatments. (h) ESR spectra of different reaction solutions with DMPO as the spin trap. (i) TEM images of MMSNs after incubation in SBF solutions at diverse pH values (7.4 or 5.5) and GSH concentrations (0 or 10 mM) for different time intervals. Scale bar, 200 nm. (j) T_1_-weighted MR images of MMSNs under different pH and GSH conditions and (k) corresponding T_1_^-1^ versus Mn concentration in MMSNs.

The monodispersed mesoporous silica nanoparticles (MSNs) were firstly synthesized via a typical sol-gel method by using cetyltrimethylammonium chloride (CTAC) as the pore-forming agent (Figure S1) [10]. Interestingly, Mn-doped MSNs (MMSNs) could be directly obtained by topological transformation of MSNs into MMSNs under hydrothermal condition [37]. The well-defined dispersity and uniform spherical morphology of MMSNs could be clearly observed from the transmission electron microscopy (TEM) (Figures 2(a) and 2(b)) and scanning electron microscopy (SEM) images (Figure S2(a)). Moreover, MMSNs exhibited the well-defined mesoporous structure with a Brunauer-Emmett-Teller (BET) surface area of 182 m^2^ g^-1^, a pore volume of 0.46 cm^3^ g^-1^, and an average pore size of 3.93 nm (Figures 2(c) and 2(d)). The successful engineering of Mn elements within the framework was verified by energy-dispersive spectrum (EDS) (Figure S2(b)) and corresponding element mapping (Figure 2(e)). The valence state of Mn was further measured by X-ray photoelectron spectroscopy (XPS). The result displayed that the ratio of Mn^2+^, Mn^3+^, and Mn^4+^ in MMSNs was calculated to be 17.8%, 44.3%, and 37.9%, respectively (Figure 2(f)). Large amounts of Mn^3+^ and Mn^4+^ contributed to the redox reaction with endogenous GSH as well as enhanced biodegradation and Fenton-like effect.

To confirm the Fenton-like activity of MMSNs, methylene blue (MB) was employed as the indicator to examine the production of hydroxyl radicals (∙OH) in the bicarbonate buffer. As shown in Figure 2(g), the absorbance of MB was notably diminished after incubation with MMSNs and H_2_O_2_ under the acidic condition, which would be ascribed to the dissociation of MMSNs into Mn^2+^ at the acidic pH and the accompanied Fenton-like reaction between Mn^2+^ and H_2_O_2_. Remarkably, MB had almost completely degraded when treated with MMSNs and H_2_O_2_ under reducing condition with or without acidic pH, revealing that a large amount of Mn^3+^ and Mn^4+^ existing in the framework of MMSNs would be reduced to Mn^2+^ by GSH and subsequently propelled Mn^2+^-mediated Fenton-like reaction for generating ∙OH. The electron spin resonance (ESR) spectra using 5,5-dimethyl-1-pyrroline-N-oxide (DMPO) as the ∙OH trapping agent presented the characteristic 1 : 2 : 2 : 1 signal in the presence of MMSNs and H_2_O_2_ (Figure 2(h)). Moreover, the signal intensity of ∙OH characteristic peak could be dramatically intensified when adding acidic pH and/or reducing GSH, which showed a similar trend with the MB degradation assay. These results illustrated that the ∙OH generation ability of MMSNs would be significantly enhanced with the assistance of acidic pH and reducing GSH due to the effective reduction and release of Mn^2+^ in these conditions.

The specific degradation of MMSNs in reducing pH values not only contributed to the substantial reduction and release of Mn^2+^ for highly efficient Fenton-like reaction but also guaranteed the excellent biocompatibility and T_1_-weighted MR imaging of nanocarrier. Hence, the biodegradable behavior of MMSNs was systematically investigated by incubating MMSNs with simulated body fluid (SBF) at diverse pH values (7.4 or 5.5) and GSH concentrations (0 or 10 mM), followed by observing the structural evolution via the TEM imaging technique. As shown in Figure 2(i), the spherical morphology and structure of MMSNs remained unchanged in neutral SBF solution without GSH for 72 h. Nevertheless, the notable structural collapse and dissolution of MMSNs were observed when exposing to the acidic or reducing SBF solutions. Importantly, the framework of MMSNs could rapidly disintegrate after immersion in the mildly acidic (pH=5.5) SBF solution containing GSH (10 mM) for 72 h, stating the high sensitivity/biodegradation of MMSNs to acidic and reducing TME. Subsequently, we examined the in vitro TME-activated T_1_-weighted MR imaging since more Mn^2+^ paramagnetic centers would become accessible to water molecules after the degradation of MMSNs. The remarkable concentration-dependent brightening effect of T_1_-weighted MR images was verified after incubating MMSNs in SBF solutions at diverse pH values (7.4 or 5.5) and different GSH concentrations (0 or 10 mM) (Figure 2(j)). The longitudinal relaxivity ($r_{1}$) of MMSNs was determined to be 5.88 mM^-1^ s^-1^ or 8.56 mM^-1^ s^-1^ in neutral (pH=7.4) or mildly acidic (pH=5.5) solution, respectively, which would be increased to 10.82 mM^-1^ s^-1^ or 13.05 mM^-1^ s^-1^, respectively, after the addition of GSH (10 mM), stating the significantly enhanced release of Mn^2+^ from MMSNs and the prominently elevated interaction between Mn^2+^ and water protons under mildly acidic and reducing conditions.

### 2.2. Construction and Intracellular ROS Generation of GR@MMSNs-P

It is well known that the effective enzymatic protection and the successful endo-/lysosomal escape of cargo gene complexes are the key factors for increased gene delivery efficacy [38–41]. Photoinduced ROS has been demonstrated to be highly effective in overcoming endo-/lysosomal sequestration [17, 42, 43]. However, limited by the shallow laser penetration depth, we herein proposed TME-triggered destruction of endo-/lysosomal membrane by ROS generated from the activated endogenous substances. To construct the competent TMCI-mediated gene delivery vectors, MMSNs were firstly aminated via the typical silanization (MMSNs-NH_2_), followed by an amidation reaction with succinic acid to obtain MMSNs-COOH. Afterwards, MMSNs-P could be obtained by the covalent conjugation between MMSNs-COOH and polyethyleneimine (PEI). The employed molecular weight of PEI was 5000, which guaranteed the low toxicity and high intracellular uptake of MMSNs-P. Ultimately, the GR@MMSNs-P complexes were acquired by encapsulating GOx and siRNA step by step (Figure 1(a)).

**Figure 3:**
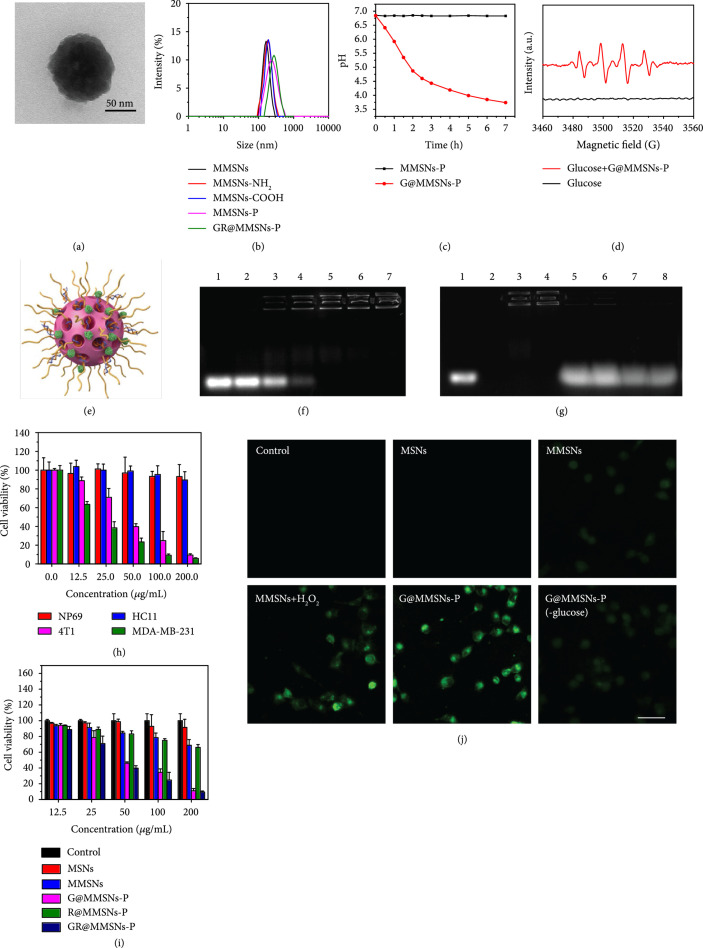
Construction and intracellular ROS production of GR@MMSNs-P. (a) TEM image of GR@MMSNs-P. (b) Hydrodynamic diameters of MMSNs, MMSNs-NH_2_, MMSNs-COOH, MMSNs-P, and GR@MMSNs-P. (c) pH values in the glucose solutions with the addition of MMSNs-P and G@MMSNs-P. (d) ESR spectra of glucose solution with or without the addition of G@MMSNs-P. (e) Schematic diagram of GR@MMSNs-P. (f) Gel retardation electrophoresis of GR@MMSNs-P complexes. Lane 1: naked siRNA; lanes 2-7: complex nanoparticles fabricated at different MRs of 1, 5, 10, 15, 20, and 30, respectively. (g) RNase protection assay. Lane 1: naked siRNA; lane 2: naked siRNA+RNase A; lane 3: GR@MMSNs-P (MR=15); lane 4: GR@MMSNs-P (MR=30); lane 5: GR@MMSNs-P+heparin (MR=15); lane 6: GR@MMSNs-P+heparin (MR=30); lane 7: GR@MMSNs-P+RNase A+heparin (MR=15); lane 8: GR@MMSNs-P+RNase A+heparin (MR=30). (h) Relative cell viabilities of different cell lines after treatment with varied concentrations of GR@MMSNs-P in 48 h of incubation. (i) Relative cell viabilities of 4T1 cells after incubation with MSNs, MMSNs, G@MMSNs-P, R@MMSNs-P, and GR@MMSNs-P at different concentrations in 48 h of incubation. (j) Intracellular ROS level after treatment with MSNs, MMSNs, MMSNs+H_2_O_2_, G@MMSNs-P, and G@MMSNs-P under low-glucose DMEM medium. Scale bar, 50 *μ*m.

The successful functionalization of MMSNs was verified by the TEM imaging technique (Figure 3(a)), the slight increase in particulate size (Figure 3(b)), the notable change in zeta potential (Figure S4), the characteristic peaks in XPS spectra (Figure S3(a)), and the remarkable weight loss in thermogravimetric analysis (TGA, Figure S3(b)). Here, the loading capacity of GOx was determined to be 3.7% via the BCA protein assay. To assess the catalytic activity of G@MMSNs-P, glucose was employed to mimic the glycolysis reaction since GOx can catalyze the oxidization of glucose into gluconic acid and H_2_O_2_. Compared to the constant pH value of MMSNs-P, the remarkable pH decline was witnessed after treatment with G@MMSNs-P over time, stating the high catalytic activity of GOx though being encapsulated on nanoparticles (Figure 3(c)). ESR spectra further confirmed the potent generation of ∙OH driven by the Fenton-like reaction between G@MMSNs-P and H_2_O_2_ produced (Figure 3(d)). The gel retardation assay was subsequently performed to investigate siRNA loading capacity of G@MMSNs-P (Figure 3(f)). The result displayed that with the increased concentration of G@MMSNs-P, their condensing ability enhanced. When the nanovector/siRNA mass ratio (MR) was 15 and above, the stable complexes would be formed between G@MMSNs-P and siRNA, and the siRNA loading amount of G@MMSNs-P was calculated to be 66.7 *μ*g mg^-1^ at MR of 15. Moreover, the RNase protection assay was further executed to assess whether G@MMSNs-P could protect siRNA from enzyme degradation (Figure 3(g)). Obviously, naked siRNA could be completely degraded by the RNase and not be detected on the gel (lanes 1 and 2). siRNA trapped in G@MMSNs-P could hardly be released from the nanovectors while it dissociated with the assistance of negatively charged heparin (lanes 3-6). Lanes 7 and 8 demonstrated that RNase could not induce siRNA degradation under the protection of G@MMSNs-P. To evaluate whether the produced ∙OH would damage siRNA, we incubated GR@MMSNs-P in the simulated TME (pH=5.5, $\left[\text{GSH}\right]=10\text{mM}$) overnight and conducted the heparin competitive displacement assay. As shown in Figure S5, siRNA could scarcely release from G@MMSNs-P (lanes 1-2) while it appeared under the introduction of negatively charged heparin (lane 3). Lanes 4-5 indicated that siRNA in G@MMSNs-P could maintain the integrity without detectable damage in the simulated TME, illustrating that the ∙OH generated from TMCI would not destroy the stability of siRNA. Moreover, GR@MMSNs-P showed good colloidal stability in DMEM medium and 10% FBS over a period of 3 days (Figures S6 and S7).

To assess the intracellular gene delivery efficacy of GR@MMSNs-P, siRNA was labeled with the fluorescent dye carboxyfluorescein (FAM). The highly metastatic 4T1 breast cancer cells were preincubated with GR@MMSNs-P for 0.5, 2, and 6 h and subsequently visualized using confocal laser scanning microscopy (CLSM) as well as quantified with flow cytometry (Figures S8(a) and S8(b)). Compared to free siRNA, the green fluorescence signals in GR@MMSNs-P strengthened with the prolonged incubation time, stating that GR@MMSNs-P complexes could be effectively internalized by 4T1 cells. Encouraged by the outstanding catalytic performance of MMSNs, the therapeutic effect of GR@MMSNs-P against 4T1 cancer cells was assessed by the typical Cell Counting Kit-8 (CCK-8) assay. Compared to normal cells, tumor cells have acidic pH and reducing microenvironment and are more sensitive to ROS-induced cell death [44]. The cell viability assay proved that GR@MMSNs-P exhibited negligible cytotoxicity against both the normal nasopharyngeal cell line NP69 and mammary epithelium cell line HC11 while exhibiting significant dose-dependent cytotoxicity to 4T1 and MDA-MB-231 cancer cells, which was attributed to the excessive accumulation of highly toxic ∙OH in cancer cells (Figure 3(h)). Interestingly, MMSNs and R@MMSNs-P also showed a dose-dependent cytotoxicity to 4T1 cells while G@MMSNs-P and GR@MMSNs-P presented the stronger cytotoxicity (Figure 3(i)), illustrating that the consumed glucose by GOx could significantly decrease cell viability and the concomitant H_2_O_2_ would be substantially converted into ∙OH by Mn^2+^-mediated Fenton-like reaction [21, 30]. To intuitively observe the intracellular ROS level, 4T1 cells were stained with the ROS fluorescence probe 2,7-dichlorofluorescin diacetate (DCFH-DA) after incubation with MSNs, MMSNs, MMSNs with the addition of H_2_O_2_, G@MMSNs-P, and G@MMSNs-P under low-glucose DMEM medium. As revealed in Figure 3(j) and Figure S9, the green fluorescence was scarcely observed in control and MSNs groups while it was slightly enhanced in the MMSNs group, which was because the low endogenous H_2_O_2_ in 4T1 cells would be catalyzed by Mn^2+^ in the framework of MMSNs. Remarkably, the green fluorescence could be significantly elevated in the cells incubated with MMSNs+H_2_O_2_ and G@MMSNs-P, stating the tremendous ROS production enhanced by H_2_O_2_. Comparatively, the cells treated with G@MMSNs-P under low-glucose DMEM medium displayed much weaker fluorescence than those under high-glucose DMEM medium, indicating the importance of glucose nutrient in exerting GOx-involved Fenton-like-mediated anticancer performance.

### 2.3. Endo-/Lysosomal Disruption Capability

**Figure 4:**
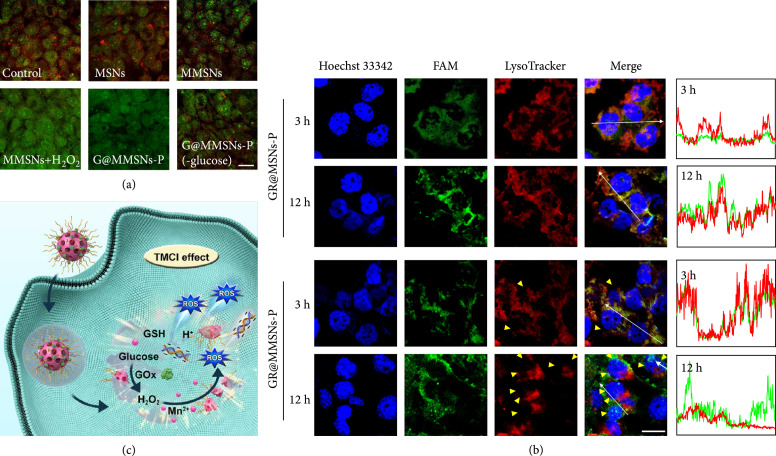
Endo-/lysosomal disruption capability of GR@MMSNs-P. (a) CLSM images of 4T1 cells after incubation with MSNs, MMSNs, MMSNs+H_2_O_2_, G@MMSNs-P, and G@MMSNs-P under low-glucose DMEM medium for 12 h, followed by staining with AO. Lysosome membrane permeabilization was determined by the decreased AO red fluorescence. Scale bar, 25 *μ*m. (b) Endo-/lysosomal escape behavior determined by CLSM images of 4T1 cells treated with GR@MSNs-P and GR@MMSNs-P for 3 and 12 h. FAM-labeled siRNA was shown in green. Lysosome was stained with LysoTracker™ Deep Red. Yellow triangles represented the withered endo-/lysosomes. Scale bar, 20 *μ*m. (c) Schematic illustration of the endo-/lysosomal escape of GR@MMSNs-P.

To investigate whether the intracellular ROS could disrupt the endo-/lysosomal membrane, the integrity of the lysosomal membrane was revealed by the acridine orange (AO) which emits red fluorescence in the acidic lysosome and green fluorescence in the cytosol and nucleus [18]. As shown in Figure 4(a), the red and green fluorescence was emitted from 4T1 cells incubated with MSNs, suggesting the integrated lysosomal membrane. However, the red fluorescence was declined when 4T1 cells were treated with MMSNs and G@MMSNs-P under low-glucose DMEM medium while it was scarcely observed in the cells incubated with MMSNs+H_2_O_2_ and G@MMSNs-P, which was consistent with the intracellular ROS generation, further confirming that the Fenton-like-mediated ∙OH could damage the lysosomal membrane structure and increase lysosomal membrane permeation.

To verify ROS-assisted TMCI for endo-/lysosomal escape capability, the colocalization of cargo gene complexes (green) and endo-/lysosomes (red) was directly observed using CLSM. After 3 h incubation with 4T1 cancer cells, most of GR@MSNs-P and GR@MMSNs-P were both entrapped in the endo-/lysosomes, which was demonstrated by the highly overlapping green and red fluorescence (Figure 4(b)). However, when the incubation time was extended to 12 h, the green and red fluorescence was still overlapped in the cells treated with GR@MSNs-P while being mostly isolated for GR@MMSNs-P, suggesting that the ∙OH generated from GR@MMSNs-P in TME could disrupt the integrated lysosomal membrane structure and assist intracellular siRNA complexes to escape from endo-/lysosomes. These above results demonstrated that TME-induced Fenton-like-mediated ROS formation could cause endo-/lysosomal destruction with translocation of the internalized cargo into the cytosol for interacting with its designated target (Figure 4(c)).

### 2.4. In Vitro Antimetastatic Effects

**Figure 5:**
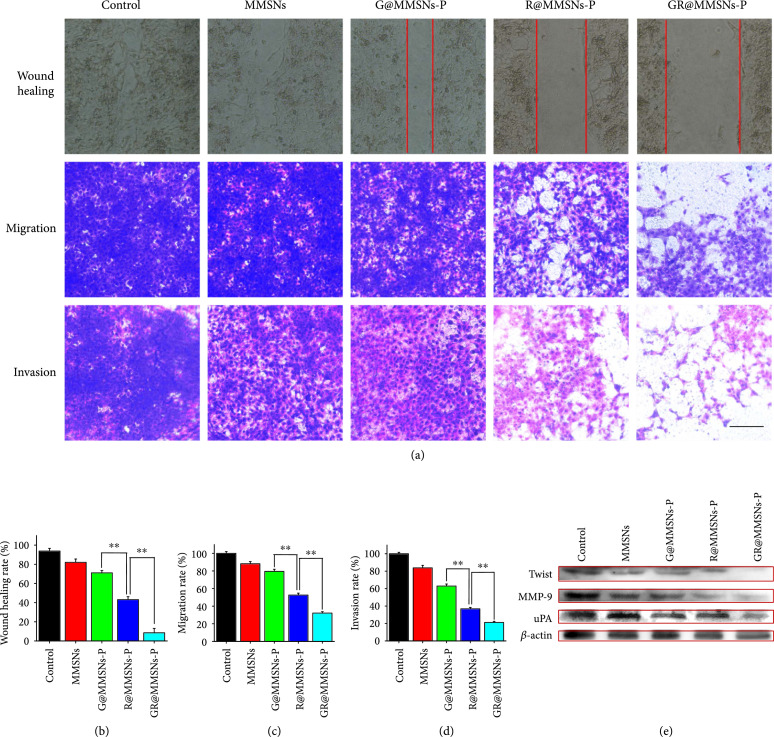
In vitro antimetastatic effects of GR@MMSNs-P. (a) Microscopy images and corresponding quantitative analysis of (b) wound healing, (c) migration, and (d) invasion of 4T1 cells after treatment with MMSNs, G@MMSNs-P, R@MMSNs-P, and GR@MMSNs-P. Scale bar, 100 *μ*m. Data were given as mean±SD (n=3). **P < 0.01. (e) The expression of Twist, MMP-9, and uPA proteins in 4T1 cells treated with MMSNs, G@MMSNs-P, R@MMSNs-P, and GR@MMSNs-P.

The transcription factor Twist has been proven to induce the loss of E-cadherin-mediated cell-cell adhesion and the expression of mesenchymal markers in epithelial cells [31, 32], thus promoting EMT and tumor metastasis [45–47]. Elimination of Twist expression in highly metastatic mammary carcinoma cells could significantly inhibit their metastasis from the mammary gland to the lung [36]. To investigate the inhibitory capability of GR@MMSNs-P on cell motility and cell interaction, the wound healing assay was firstly implemented. The cells in the control group presented the strong scratch healing ability with the healing rate of 93.85% at 24 h after scratching, demonstrating the highly metastatic characteristic of 4T1 cancer cells. The cells treated with MMSNs and G@MMSNs-P revealed the slight inhibitory effects with the healing rate of 82.10% and 71.08%, respectively, which might be attributed to the produced ROS in the cells. Interestingly, anti-Twist siRNA-containing MMSNs-P exhibited stronger migration inhibitory ability with the healing rate of 43.13% than the nanovectors without siRNA, suggesting the tremendous downregulation of Twist protein. Moreover, the lowest healing rate of 8.52% was obtained in the cells treated with GOx- and siRNA-coloaded MMSNs, illustrating the prominent synergistic inhibition effect between GOx, anti-Twist siRNA, and MMSNs on cell mobility (Figures 5(a) and 5(b)).

The biological effects of GR@MMSNs-P on the longitudinally migratory and invasive behaviors of 4T1 cells were systematically investigated by using the transwell chamber assay with or without the Matrigel-coated membrane, respectively. The qualitative and quantitative results showed a similar trend to that of the wound healing assay (Figures 5(a), 5(c), and 5(d)). Compared to the control groups, MMSNs and G@MMSNs-P groups exhibited mild inhibition effect on migration capability of 4T1 cells with the coverage rates of 88.20% and 79.51% on the lower surface in the migration assay, as well as 83.83% and 62.96% in the invasion assay, respectively. The number of invaded cells into the lower chamber was obviously reduced to 52.62% and 36.87% in the R@MMSNs-P group for the migration and invasion assays, respectively, which further reduced to 32.13% and 21.22%, respectively, in the GR@MMSNs-P group, illustrating the essential roles of anti-Twist siRNA and synergistic intracellular ROS in inhibiting tumor invasion and metastasis. To further explore the possible mechanisms contributing to the remarkable anti-migration and anti-invasion effects of GR@MMSNs-P, three representative metastasis-promoting markers in 4T1 cells including Twist, matrix metalloproteinase-9 (MMP-9, an endopeptidase capable of degrading extracellular matrix), and urokinase-type plasminogen activator (uPA, an extracellular proteinase) were detected using the immunoblotting assay. As shown in Figure 5(e), 4T1 cells in the control group displayed high expression of Twist, MMP-9, and uPA, whereas the expression of all these proteins was slightly downregulated after treatment with MMSNs, G@MMSNs-P, or R@MMSNs-P. Meanwhile, the strongest inhibitory effect on the expression of Twist, MMP-9, and uPA was witnessed in the GR@MMSNs-P group, implying the excellent synergistic anti-metastatic effect between MMSNs, GOx, and anti-Twist siRNA.

### 2.5. In Vivo Biodistribution

**Figure 6:**
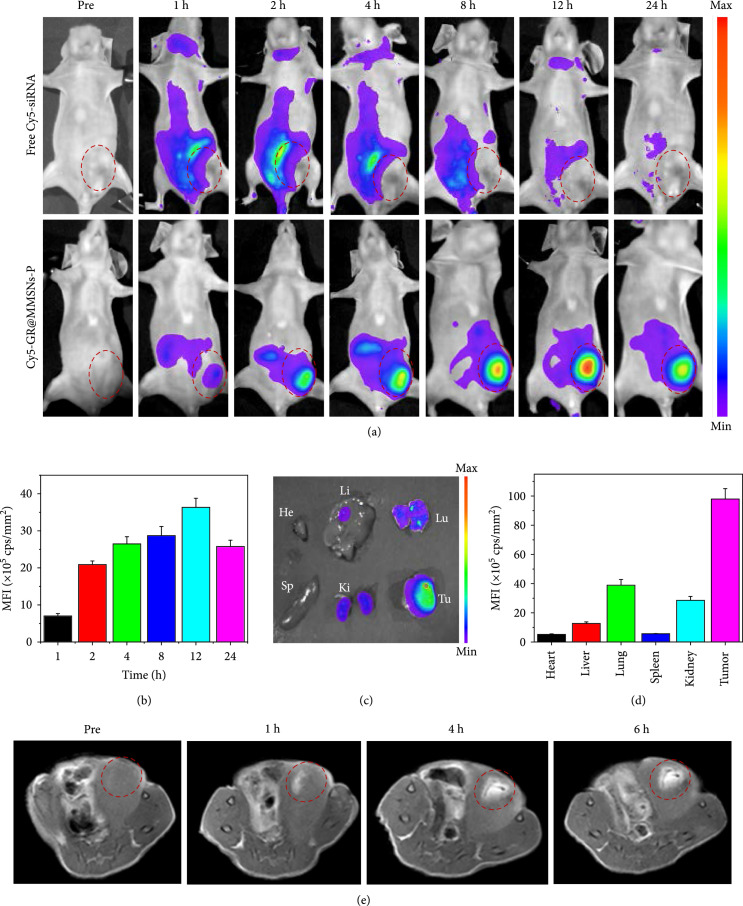
In vivo biodistribution and MR imaging of GR@MMSNs-P. (a) In vivo fluorescence images of the mice bearing the 4T1 tumor after intravenous administration of free Cy5-siRNA and Cy5-GR@MMSNs-P at different time points and (b) the corresponding mean fluorescence intensity (MFI) values. (c) Ex vivo fluorescence images of major organs and tumor tissue harvested at 24 h postinjection. He: heart; Li: liver; Sp: spleen; Lu: lung; Ki: kidney; Tu: tumor. (d) Corresponding MFI values of ex vivo tissues at 24 h postinjection. (e) In vivo T_1_-weighted MR images of the mice bearing the 4T1 tumor before and after intravenous injection of GR@MMSNs-P at 0, 1, 4, and 6 h.

To evaluate the in vivo biodistribution of GR@MMSNs-P, siRNA was fluorescently labeled with Cy5 dye and tracked using an In Vivo Imaging System (IVIS) on mice bearing 4T1 breast cancer (Figures 6(a) and 6(b)). Compared to free Cy5-siRNA, much more obvious fluorescent signals clearly appeared in the tumor region after intravenous administration of Cy5-GR@MMSNs-P. Moreover, the fluorescence intensity in the tumor site increased with time extension and reached its maximum at 12 h postinjection, stating the efficient tumor accumulation of those GR@MMSNs-P. The ex vivo fluorescence image further confirmed that GR@MMSNs-P were mostly distributed in the tumor tissue which showed the highest fluorescence in comparison with those major organs, such as the heart, liver, spleen, lung, and kidney (Figures 6(c) and 6(d)). The specific degradation of MMSNs in reducing pH values has been demonstrated to be conducive to the release of Mn^2+^ for enhanced T_1_-MR imaging contrast in vitro. To further confirm the in vivo MR imaging performance of GR@MMSNs-P for tumor-specific diagnosis, they were intravenously administrated into the mice bearing the 4T1 tumor. The T_1_-weighted MR imaging signal intensified over time in the tumor site, indicating that GR@MMSNs-P could be efficiently accumulated in the tumor area and gradually degraded and reduced to Mn^2+^ ions for enhancing the accessibility of water molecules to Mn^2+^ paramagnetic centers, thus realizing TME-activated T_1_-weighted MR imaging contrast effect (Figure 6(e)).

### 2.6. In Vivo Therapeutic Efficacies

**Figure 7:**
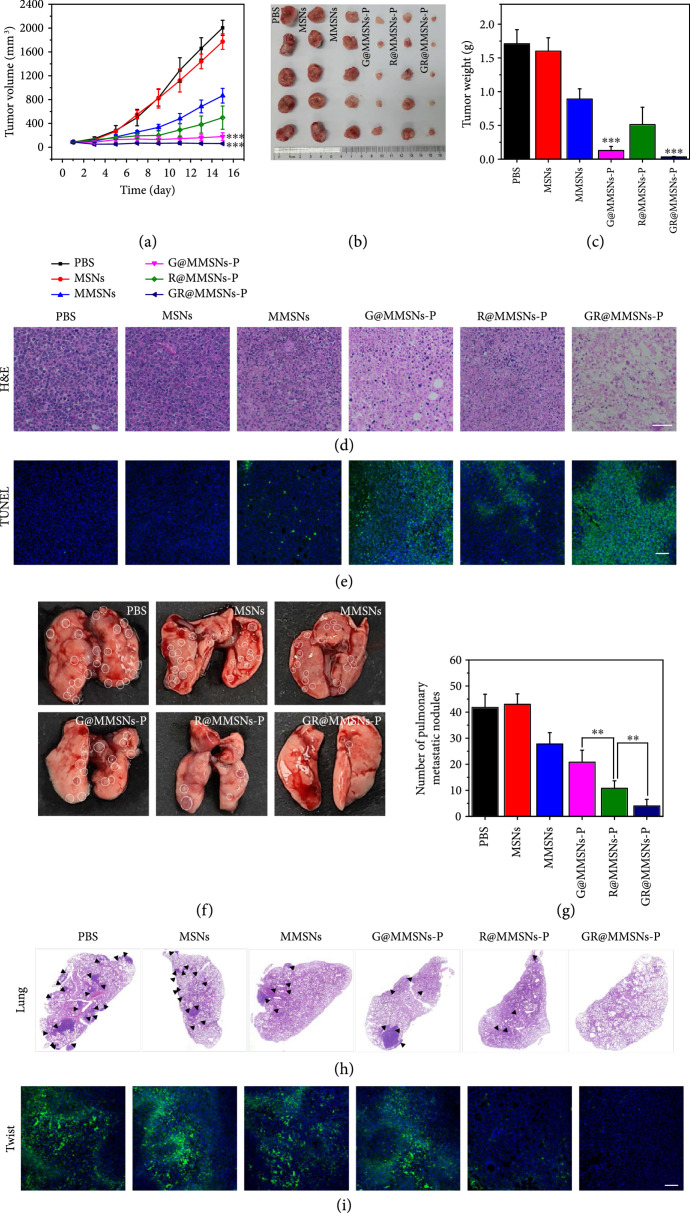
In vivo therapeutic efficacies. (a) Tumor growth curves of the mice bearing the 4T1 tumor during the experiment. (b) Photographs and (c) weights of the tumors removed from the mice bearing the 4T1 tumor in different groups at the end of treatment. (d) H&E staining (scale bar, 50 *μ*m) and (e) TUNEL assay (scale bar, 100 *μ*m) of tumor tissues at the end of treatment. (f) Representative photographs and (h) H&E-stained lung tissues of 4T1 tumor-bearing mice in different treatment groups. (g) Quantitative analysis of pulmonary metastatic nodules in different groups. (i) The immunofluorescence analysis of Twist protein expressions in primary tumors with the different treatments. Scale bar, 100 *μ*m. All statistical data were presented as means±SD (n=5). **P < 0.01 and ***P < 0.001.

The in vivo inhibitory effects against breast cancer growth and metastasis were assessed using the mice bearing the 4T1 tumor. All the mice were randomly assigned into six groups and intravenously administrated with PBS, MSNs, MMSNs, G@MMSNs-P, R@MMSNs-P, or GR@MMSNs-P. Then, the tumor volumes and body weights of all groups were recorded every 2 days during the whole period of treatment. The mice injected with MMSNs and R@MMSNs-P exhibited limited inhibition on tumor growth due to the Fenton-like effect of MMSNs in TME with certain H_2_O_2_ and acidity. Comparatively, G@MMSNs-P and GR@MMSNs-P groups showed the most significant tumor inhibition effects through the cascaded catalytic reactions involving GOx-mediated glucose depletion and Mn^2+^-mediated ROS generation (Figures 7(a)–7(c) and S10). The hematoxylin and eosin (H&E) and terminal deoxynucleotidyl transferase-mediated dUTP nick end labeling (TUNEL) staining of tumor slices further confirmed the maximum tumor cell destruction and apoptosis in the GR@MMSNs-P group (Figures 7(d) and 7(e)). In addition, in comparison with the control group, no obvious pathological changes of major organs (heart, liver, kidney, and spleen) and body weight variations were observed in all treatment groups, stating the excellent biocompatibility and negligible systemic toxicity of these treatments (Figures S11 and S12).

The anti-metastasis effect was next assessed by the number and H&E staining of metastatic nodules in the lung tissues (Figures 7(f)–7(h)), and in the meantime, the possible anti-metastasis mechanism was explored by the immunofluorescence assay (Figure 7(i)). It was worth noting that although the primary tumor volumes in the R@MMSNs-P group were a little larger than those in the G@MMSNs-P group, the pulmonary metastatic nodules in the R@MMSNs-P group were obviously less than those in the G@MMSNs-P group, which was attributed to the effective downregulation of Twist protein by using siRNA-loaded MMSNs. Moreover, importantly, no obvious metastasis was found in the GR@MMSNs-P treated group, and the H&E-stained images of lung sections also verified scarcely any micrometastatic foci in the inner lung tissue (Figure S13). These results illustrated that the massive glucose consumption and ROS production in tumors caused by GR@MMSNs-P could not only effectively inhibit the primary tumor growth but also significantly enhance the Twist-silencing efficiency, eventually resulting in prominent inhibition performance on breast cancer growth and pulmonary metastasis.

## 3. Discussion

TME-triggered enhanced chemical internalization was successfully realized by taking full advantage of the specific TME, which was significantly different from PCI. The constructed GR@MMSNs-P could remarkably elevate ROS production due to the cascaded catalytic reactions involving GOx-mediated redox reaction and Mn^2+^-mediated Fenton-like reaction, which was independent of exogenous laser irradiation. The excessively accumulated ROS in situ in tumor tissues would not only exert strong tumor-killing effect but also destroy the endocytic membrane, promote gene endo-/lysosomal escape, and synergistically improve gene transfection efficiency. Anti-Twist siRNA-loaded G@MMSNs-P were demonstrated to be effectively accumulated in the tumor site and resulted in suppressed tumor growth and pulmonary metastasis due to the excellent synergistic effect between GOx, anti-Twist siRNA, and MMSNs. Moreover, the nonviral vectors G@MMSNs-P were prone to degrade under the acidic and reducing TME due to the incorporated Mn in the framework of MMSNs, which endowed the nanovectors with high biocompatibility and excellent T_1_-weighted MR imaging contrast performance. TME-specific chemical internalization provided a new insight into RNA interference therapeutics with high efficiency and feasibility.

## 4. Materials and Methods

### 4.1. Materials

Cetyltrimethylammonium chloride (CTAC, 25 wt%), tetraethyl orthosilicate (TEOS), 3-aminopropyltriethoxysilane (APTES), MnSO_4_·H_2_O, glucose oxidase, and 5,5-dimethyl-1-pyrroline N-oxide (DMPO) were obtained from Sigma-Aldrich. Triethanolamine (TEA) and methylene blue (MB) were obtained from Aladdin Industrial Inc. Disodium maleate, succinic acid, 1-(3-dimethylaminopropyl)-3-ethylcarbodiimide hydrochloride (EDCI), and 1-hydroxybenzotriazole monohydrate (HOBT) were obtained from Tokyo Chemical Industry Co., Ltd. Polyethylene imine (PEI, molecular weight 5000, 50 wt%) was obtained from Shanghai Yuanye Bio-Technology Co., Ltd. Small interfering RNA against Twist (anti-Twist siRNA) (5′-GCU GAG CAA GAU UCA GAC CTT-3′ (sense)), FAM-labeled scrambled RNA (FAM siRNA), and Cy5-labeled scrambled RNA were obtained from Guangzhou Ribo Bio-Technology Co., Ltd. RNase A and RNase inhibitor were bought from the Beyotime Institute of Biotechnology. The anti-Twist, anti-beta actin, and anti-MMP-9 were purchased from Abcam Biotechnology. The anti-uPA antibody was obtained from Santa Cruz Biotechnology.

### 4.2. Synthesis of MMSNs

CTAC (2 g) and TEA (0.02 g) were dissolved in deionized water (18 mL) in turn at 80°C and stirred for 20 min. The solution containing TEOS (1.4 g) was added dropwise, and the resultant mixture continued to be stirred for 4 h. Then, the products were obtained by centrifugation and washing with ethanol. The solution of sodium chloride (NaCl) in methanol (8 mg mL^-1^) was employed to remove the template CTAC by means of extraction. Subsequently, the obtained MSNs were dispersed into a mixture solution (10 mL) containing MnSO_4_·H_2_O (16 mg mL^-1^) and disodium maleate (20 mg mL^-1^). Finally, the mixture was treated under hydrothermal condition at 180°C for 48 h. And the products were obtained by centrifugation.

### 4.3. Synthesis of MMSNs-P

The MMSNs prepared above were dispersed in ethanol (200 mL) and refluxed for 12 h at 78°C, followed by adding APTES (1 mL). After centrifugation and washing, MMSNs-NH_2_ were redispersed in ethanol (8 mL) and added into the solution of succinic acid (100 mg), EDCI (130 mg), and HOBT (130 mg) in ethanol (2 mL). After stirring for 12 h, the obtained MMSNs-COOH were centrifuged, washed repeatedly, and dispersed in ethanol (5 mL). To covalently graft PEI onto the surface of MMSNs-COOH, MMSNs-COOH (5 mL), EDCI (130 mg), and HOBT (130 mg) were ultrasonically treated for 30 min and then added dropwise into the solution containing PEI (120 mg, 50 wt%, 2 mL). Ultimately, the mixture was stirred for 48 h, centrifuged, and washed repeatedly with ethanol and water.

### 4.4. Preparation of GR@MMSNs-P

G@MMSNs-P were prepared by incubating MMSNs-P with the GOx solution (1 mg mL^-1^, 5 mL) at room temperature for 24 h and subsequently centrifuging to remove free GOx. Anti-Twist siRNA (0.2 mg mL^-1^) was added into the G@MMSNs-P solution (200 *μ*L) with different concentrations, and the resulting mixtures were vortexed for 30 s and incubated for another 30 min to obtain GR@MMSNs-P.

### 4.5. Fenton-Like Activity of MMSNs

MMSNs (Mn concentration of 0.5 mM) were dispersed in NaHCO_3_ solution (25 mM) at diverse pH values (7.4 or 5.5) and GSH concentrations (0 or 1 mM). Then, the mixture was shaken at 37°C overnight. After centrifugation, H_2_O_2_ (10 mM) and MB (10 *μ*g mL^-1^) were added to the supernatant. The absorbance of MB at 664 nm was monitored by using a UV-vis instrument. To confirm the generation of ∙OH, electron spin resonance (ESR) spectra were acquired using DMPO as the ∙OH trapping agent in a perpendicular mode on a Bruker A300 spectrometer.

### 4.6. In Vitro Degradation Experiment

MMSNs (1 mg) were dispersed into simulated body fluid (SBF, 10 mL) at diverse pH values (7.4 or 5.5) and GSH concentrations (0 or 10 mM). The testing solutions were incubated in a water bath at 37°C, and a small amount of solution (100 *μ*L) was taken out at different time points for centrifugation. The precipitate was redispersed in ethanol and observed by TEM.

### 4.7. Catalytic Activity Measurements of G@MMSNs-P

To detect the catalytic activity of GOx in G@MMSNs-P, G@MMSNs-P (100 *μ*g/mL) and MMSNs-P (100 *μ*g/mL) were added to glucose (1 mg/mL) solutions, respectively. Then, pH values were measured with a pH meter. ESR spectroscopy was used to further determine whether the presence of GOx promoted the production of ∙OH.

### 4.8. Agarose Gel Retardation Assay

The capability of G@MMSNs-P to compress siRNA was evaluated by the agarose gel retardation assay. Anti-Twist siRNA (0.4 *μ*g) was added into G@MMSNs-P at different mass ratios, and the mixtures were loaded onto agarose gel in Tris-acetate-EDTA (TAE) with loading buffer. The electrophoresis was performed at 80 V for 25 min and imaged with the Bio-Rad imaging system (USA).

### 4.9. RNase A Protection Assay

Naked siRNA and GR@MMSNs-P solutions containing 0.4 *μ*g siRNA with different mass ratios were incubated with 50 ng RNase A at 37°C for 10 min. Subsequently, RNase inhibitor and heparin solution (150 mg mL^-1^, 5 *μ*L) were added into the above solutions in sequence. The mixtures were incubated at 50°C for 4 h. The release of siRNA from the complexes was assessed by using agarose gel electrophoresis.

### 4.10. Evaluation of siRNA Stability in the Simulated TME

GR@MMSNs-P containing 0.4 *μ*g siRNA were incubated in the SBF solutions with pH value (5.5), GSH concentration (10 mM), and glucose (1 mM) at 37°C overnight. Then, the samples were centrifuged and the precipitates were redispersed in water. Subsequently, the obtained solutions were treated with heparin solution before agarose gel electrophoresis.

### 4.11. Cellular Uptake

4T1 cells were seeded in the confocal dish ($2\times 10^{5}$ cells/dish) and treated with FAM-siRNA loaded G@MMSNs-P (FAM-siRNA: 1.25 *μ*g mL^-1^ per well) for 0.5, 2, and 6 h at 37°C. Afterwards, the cells were washed with PBS for three times and fixed with paraformaldehyde (4%) solution, followed by staining the nuclei with 4′,6-diamidino-2-phenylindole (DAPI). The intracellular fluorescence was observed by confocal laser scanning microscopy (CLSM, LSM880, Zeiss, Germany). For the flow cytometry assay, 4T1 cells were seeded in 6-well plates and incubated with FAM-siRNA loaded G@MMSNs-P for 0.5, 2, and 6 h.

### 4.12. In Vitro Cytotoxicity Assay

4T1 cells, NP69 cells, HC11 cells, and MDA-MD-231 cells were seeded in 96-well plates ($5\times 10^{3}$ cells/well) and treated with different concentrations of nanoparticles for 48 h. At the end of incubation, the cell culture media were removed and replaced with CCK-8 solution (100 *μ*L, $V_{\text{CCK}-8}:V_{\text{DMEM}}=1:9$). The absorbance at 450 nm was measured using a microplate spectrophotometer (Epoch 2, BioTek, USA).

### 4.13. Intracellular ROS Detection

4T1 cells were incubated with MSNs, MMSNs, MMSNs+H_2_O_2_, G@MMSNs-P, or G@MMSNs-P under low-glucose DMEM medium (100 *μ*g mL^-1^) for 12 h. After that, the cells were washed and stained with DCFH-DA (10 *μ*M) for 20 min. Ultimately, intracellular fluorescence was observed using CLSM.

### 4.14. Endo-/Lysosomal Disruption

For the assessment of the integrity of the lysosomal membrane, 4T1 cells were seeded in the confocal dish ($2\times 10^{5}$ cells/dish) and treated with MSNs, MMSNs, MMSNs+H_2_O_2_, G@MMSNs-P, or G@MMSNs-P under low-glucose DMEM medium (100 *μ*g mL^-1^) for 12 h. Subsequently, the cells were stained with acridine orange (AO, 5 *μ*M) for 15 min and washed with PBS. Then, the cells were observed under CLSM. The excitation of AO was 488 nm, and the emission was 525 nm for green and 625 nm for red, respectively. For the detection of endo-/lysosomal escape behavior, 4T1 cells were treated with GR@MSNs-P and GR@MMSNs-P for 3 and 12 h, respectively. Then, the cells were stained with Hoechst 33342 (1 *μ*g mL^-1^) and LysoTracker™ Deep Red (50 nM) for 30 min and observed under CLSM.

### 4.15. Wound Healing Assay

4T1 cells were seeded in 12-well plates ($2\times 10^{5}$ cells/well) and incubated with MMSNs, G@MMSNs-P, R@MMSNs-P, and GR@MMSNs-P (20 *μ*g mL^-1^ of MMSNs, 1.25 *μ*g mL^-1^ of anti-Twist siRNA) for 48 h. The cell monolayers were scraped with pipette tips, washed with PBS thrice, incubated in fresh media for 24 h, and observed under a microscope.

### 4.16. In Vitro Migration and Invasion

For the migration assay, 4T1 cells were preincubated with MMSNs, G@MMSNs-P, R@MMSNs-P, and GR@MMSNs-P (20 *μ*g mL^-1^ of MMSNs, 1.25 *μ*g mL^-1^ of anti-Twist siRNA) for 48 h and then harvested, suspended in serum-free medium, and plated in the upper chambers ($2\times 10^{5}$ cells/chamber) of transwell. For the invasion assay, the upper chambers were coated with Matrigel (BD Biosciences), and then, $2\times 10^{5}$ cells were seeded. For both assays, complete medium was added in the lower chamber. After incubation for 24 h, the cells that migrated or invaded through the transwell membrane were fixed with paraformaldehyde (4%), stained with crystal violet (0.1%), and photographed under a microscope.

### 4.17. Western Blot Analysis

4T1 cells were seeded in 6-well plates ($5\times 10^{5}$ cells/well) and treated with MMSNs, G@MMSNs-P, R@MMSNs-P, and GR@MMSNs-P (20 *μ*g mL^-1^ of MMSNs, 1.25 *μ*g mL^-1^ of anti-Twist siRNA) for 48 h. The expressions of MMP-9, uPA, and Twist proteins were analyzed by western blotting.

### 4.18. Animal Model

Female BALB/c nude mice (16-18 g, 4-6 weeks old) were purchased from the Guangdong Medical Laboratory Animal Center (Foshan, China). All animal experiments were conducted according to the guidelines approved by the Institutional Animal Care and Use Committee of Sun Yat-sen University (Guangzhou, China). $1\times 10^{6}$ 4T1 cells were subcutaneously injected into the left mammary gland. When the tumor volume reached ~90 mm^3^, the mice were assigned into several groups randomly.

### 4.19. Biodistribution

The mice bearing 4T1 breast cancer were applied to in vivo fluorescence imaging and biodistribution. After intravenous injection of free Cy5-labeled siRNA and Cy5-labeled GR@MMSNs-P (1 mg kg^-1^ of siRNA per mouse), the mice were imaged by using the In Vivo Imaging System (Bruker, FX Pro, USA) (Ex/Em=650/680 nm) at different time points (0, 1, 2, 4, 8, 12, and 24 h). Then, the main organs (heart, liver, lung, spleen, and kidney) and tumors are collected and imaged.

### 4.20. MR Imaging Performance of MMSNs

MR imaging was conducted on a 3.0 T MRI scanner (United Imaging, China) with the following sequence: TR=600 ms and TE=12.6 ms. For in vitro MRI experiment, MMSNs with varied Mn concentrations (0, 0.02, 0.04, 0.06, 0.1, and 0.2 mM) were dispersed in SBF (1 mL) at diverse pH values (7.4 or 5.5) and GSH concentrations (0 or 10 mM). In vivo T_1_-weighted MR imaging signals were acquired before and 1, 4, and 6 h after intravenous injection of GR@MMSNs-P (1.5 mg kg^-1^ Mn) on 4T1 tumor-bearing mice.

### 4.21. Inhibition of Growth and Metastasis of Breast Cancer

The mice bearing the 4T1 tumor were randomly divided into six groups (n=5) and administered intravenously (injection of every three days, 3 doses) with PBS, MSNs, MMSNs, G@MMSNs-P, R@MMSNs-P, and GR@MMSNs-P (1 mg kg^-1^ of anti-Twist siRNA per mouse), respectively. The tumor volumes and body weights were recorded every 2 days for 15 days. At the end of treatment, the mice were sacrificed, and the primary tumors and lungs were collected and washed with cold PBS. The tumors were weighed and photographed, followed by fixation and sectioning to conduct hematoxylin and eosin (H&E) and terminal deoxynucleotidyl transferase-mediated dUTP nick end labeling (TUNEL) staining. Moreover, lung tissues were imaged, metastatic nodules were counted, and metastatic lesions were examined by H&E staining. The sectioned tumors were incubated with the anti-Twist antibody, followed by immunofluorescence analysis to examine Twist expression. The heart, liver, spleen, and kidney were also harvested, and we conducted H&E staining to evaluate the histopathologic toxicity to normal tissues.

## Data Availability

All data needed to evaluate the conclusions in the paper are present in the paper and/or supplementary materials. All other data supporting the findings of this study are available from the corresponding authors upon reasonable request.

## Supplementary Materials

Supplementary Materials Figure S1: (a, b) TEM images of the as-synthesized MSNs. Figure S2: (a) SEM image and (b) EDS spectrum of MMSNs. Figure S3: (a) XPS spectra and (b) TGA curves of MMSNs and MMSNs-P. Figure S4: zeta potentials of MMSNs, MMSNs-NH2, MMSNs-COOH, MMSNs-P, and GR@MMSNs-P. Figure S5: the stability of siRNA in the simulated TME determined by the gel retardation assay. N represented naked siRNA. “+” represented the group treated with simulated TME or heparin. “−” represented the group without simulated TME or heparin treatment. Figure S6: colloid stability of GR@MMSNs-P in DMEM medium with 10% FBS within 3 days. Figure S7: colloid stability of GR@MMSNs-P in 10% FBS within 3 days. Figure S8: (a) CLSM images of 4T1 cells treated with GR@MMSNs-P for 0.5, 2, and 6 h, respectively. Blue fluorescence: DAPI, green fluorescence: FAM-labelled siRNA. Scale bar, 50 *μ*m. (b) Intracellular uptake amounts of free siRNA and GR@MMSNs-P in 4T1 cells determined by flow cytometry analysis. Figure S9: mean florescence intensity determined by using the ImageJ software of intracellular ROS variation after different treatments. Figure S10: the individual tumor growth kinetics in control and treated groups (n=5). Figure S11: H&E-stained images of major organs (heart, liver, kidney, and spleen) collected from the mice at different groups. Scale bar, 100 *μ*m. Figure S12: body weights of the mice in different groups (n=5). Figure S13: the amplified H&E staining images of lung tissues collected from mice in different groups. Scale bar, 400 *μ*m.
