# Erratum to “Tumor Microenvironment-Specific Chemical Internalization for Enhanced Gene Therapy of Metastatic Breast Cancer”

**DOI:** 10.34133/research.0076

**Published:** 2023-03-15

**Authors:** Yun Zhou, Mian Yu, Changjun Tie, Yang Deng, Junqing Wang, Yunfei Yi, Fan Zhang, Chenyi Huang, Hairong Zheng, Lin Mei, Meiying Wu

**Affiliations:** ^1^ School of Pharmaceutical Sciences (Shenzhen), Sun Yat-sen University, Shenzhen 518107, China.; ^2^ Paul C. Lauterbur Research Center for Biomedical Imaging, Institute of Biomedical and Health Engineering, Shenzhen Institutes of Advanced Technology, Chinese Academy of Sciences, Shenzhen 518055, China.; ^3^ Tianjin Key Laboratory of Biomedical Materials, Key Laboratory of Biomaterials and Nanotechnology for Cancer Immunotherapy, Institute of Biomedical Engineering, Chinese Academy of Medical Sciences and Peking Union Medical College, Tianjin 300192, China.

In the Research Article “Tumor Microenvironment-Specific Chemical Internalization for Enhanced Gene Therapy of Metastatic Breast Cancer,” the authors made an error in Fig. 6A. In Fig. 6A, the image of “pre” in the “Free Cy5-siRNA” group was misused. When incorporating the final images in Fig. 6A, another image of “24 h” in the “Free Cy5-siRNA” group was inadvertently chosen as the “Pre” image, so they appear to be remarkably similar. After the error was discovered, the authors repeated the experiments that concluded that this error did not affect the results, discussion, or conclusion of this paper. Figure 6A has now been corrected in the PDF and HTML (full text).

**Fig. 6.**
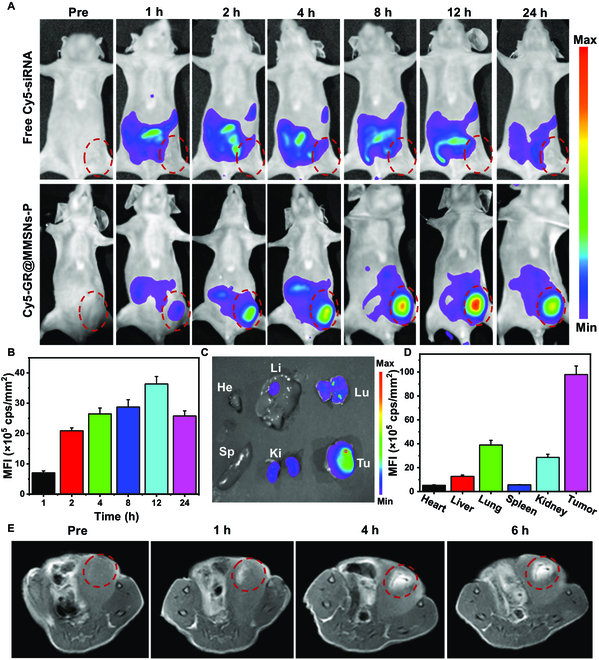
In vivo biodistribution and MR imaging of GR@MMSNs-P. (A) In vivo fluorescence images of the mice bearing the 4T1 tumor after intravenous administration of free Cy5-siRNA and Cy5-GR@MMSNs-P at different time points and (B) the corresponding mean fluorescence intensity (MFI) values. (C) Ex vivo fluorescence images of major organs and tumor tissue harvested at 24 h postinjection. He, heart; Li, liver; Sp, spleen; Lu, lung; Ki, kidney; Tu, tumor. (D) Corresponding MFI values of ex vivo tissues at 24 h postinjection. (E) In vivo T_1_-weighted MR images of the mice bearing the 4T1 tumor before and after intravenous injection of GR@MMSNs-P at 0, 1, 4, and 6 h.
