# Supplementary material for: Tumor Microenvironment-Specific Chemical Internalization for Enhanced Gene Therapy of Metastatic Breast Cancer
Source: Research (Wash D C). 2021 Jun 18;2021:9760398. doi: 10.34133/2021/9760398 (PMC11014676; doi:10.34133/2021/9760398)
Supplement: Supplementary Materials — Figure S1: (a, b) TEM images of the as-synthesized MSNs. Figure S2: (a) SEM image and (b) EDS spectrum of MMSNs. Figure S3: (a) XPS spectra and (b) TGA curves of MMSNs and MMSNs-P. Figure S4: zeta potentials of MMSNs, MMSNs-NH2, MMSNs-COOH, MMSNs-P, and GR@MMSNs-P. Figure S5: the stability of siRNA in the simulated TME determined by the gel retardation assay. N represented naked siRNA. “+” represented the group treated with simulated TME or heparin. “−” represented the group without simulated TME or heparin treatment. Figure S6: colloid stability of GR@MMSNs-P in DMEM medium with 10% FBS within 3 days. Figure S7: colloid stability of GR@MMSNs-P in 10% FBS within 3 days. Figure S8: (a) CLSM images of 4T1 cells treated with GR@MMSNs-P for 0.5, 2, and 6 h, respectively. Blue fluorescence: DAPI, green fluorescence: FAM-labelled siRNA. Scale bar, 50 μm. (b) Intracellular uptake amounts of free siRNA and GR@MMSNs-P in 4T1 cells determined by flow cytometry analysis. Figure S9: mean florescence intensity determined by using the ImageJ software of intracellular ROS variation after different treatments. Figure S10: the individual tumor growth kinetics in control and treated groups (n=5). Figure S11: H&E-stained images of major organs (heart, liver, kidney, and spleen) collected from the mice at different groups. Scale bar, 100 μm. Figure S12: body weights of the mice in different groups (n=5). Figure S13: the amplified H&E staining images of lung tissues collected from mice in different groups. Scale bar, 400 μm. [file 9760398.f1.pdf]

Research
A SCIENCE PARTNER JOURNAL
SPJ
SCIENCE PARTNER JOURNALS

Manuscript Template

Supplementary Materials

Figure S1. (a, b) TEM images of the as-synthesized MSNs.
Figure S2. (a) SEM image and (b)EDS spectrum of MMSNs.
Figure S3. (a) XPS spectra and (b) TGA curves of MMSNs and MMSNs-P.
Figure S4. Zeta potentials of MMSNs, MMSNs-NH_2, MMSNs-COOH, MMSNs-P and GR@MMSNs-P.
Figure S5. The stability of siRNA in the simulated TME determined by gel retardation assay. N represented naked siRNA. "+" represented the group treated with simulated TME or heparin. "–" represented the group without simulated TME or heparin treatment.
Figure S6. Colloid stability of GR@MMSNs-P in DMEM medium with 10% FBS within 3 days.
Figure S7. Colloid stability of GR@MMSNs-P in 10% FBS within 3 days.
Figure S8. (a) CLSM images of 4T1 cells treated with GR@MMSNs-P for 0.5, 2 and 6 h, respectively. Blue fluorescence: DAPI, green fluorescence: FAM-labelled siRNA. Scale bar, 50 μm. (b) Intracellular uptake amounts of free siRNA and GR@MMSNs-P in 4T1 cells determined by flow cytometry analysis.
Figure S9. Mean florescence intensity determined by ImageJ software of intracellular ROS variation after different treatments.
Figure S10. The individual tumor growth kinetics in control and treated groups (n = 5).
Figure S11. H&E-stained images of major organs (heart, liver, kidney and spleen) collected from the mice at different groups. Scale bar, 100 μm.
Figure S12. Body weights of the mice in different groups (n = 5).
Figure S13. The amplified H&E staining images of lung tissues collected from mice in different groups. Scale bar, 400 μm.

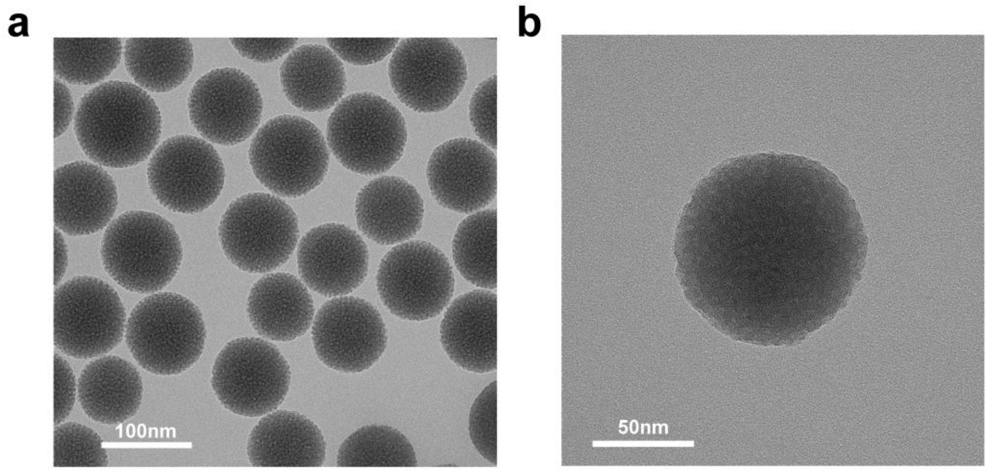

Figure S1. (a,b) TEM images of the as-synthesized MSNs.

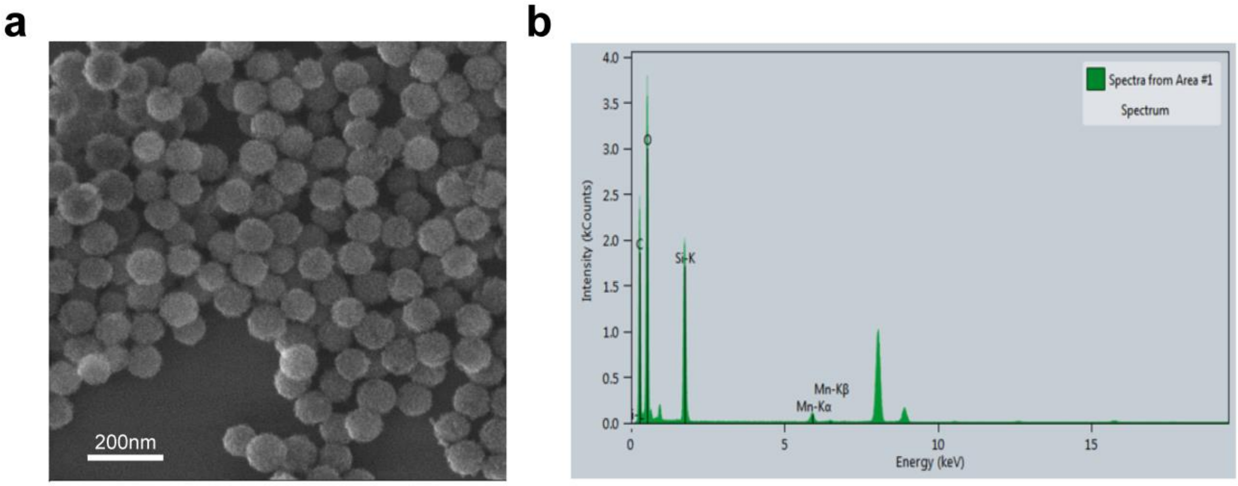

Figure S2. (a) SEM image and (b)EDS spectrum of MMSNs.

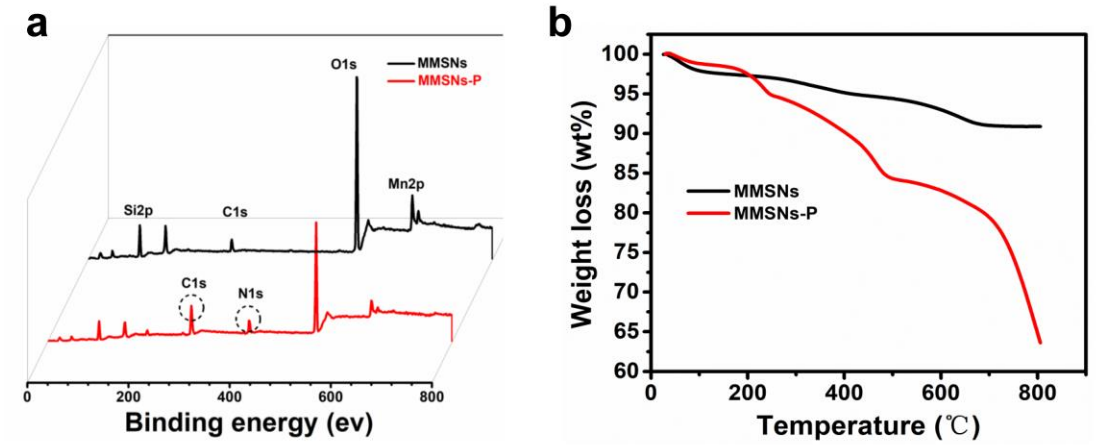

Figure S3. (a) XPS spectra and (b) TGA curves of MMSNs and MMSNs-P.

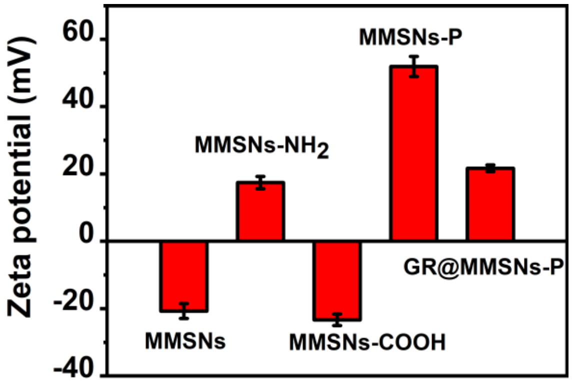

Figure S4. Zeta potentials of MMSNs, MMSNs-NH_2, MMSNs-COOH, MMSNs-P and GR@MMSNs-P.

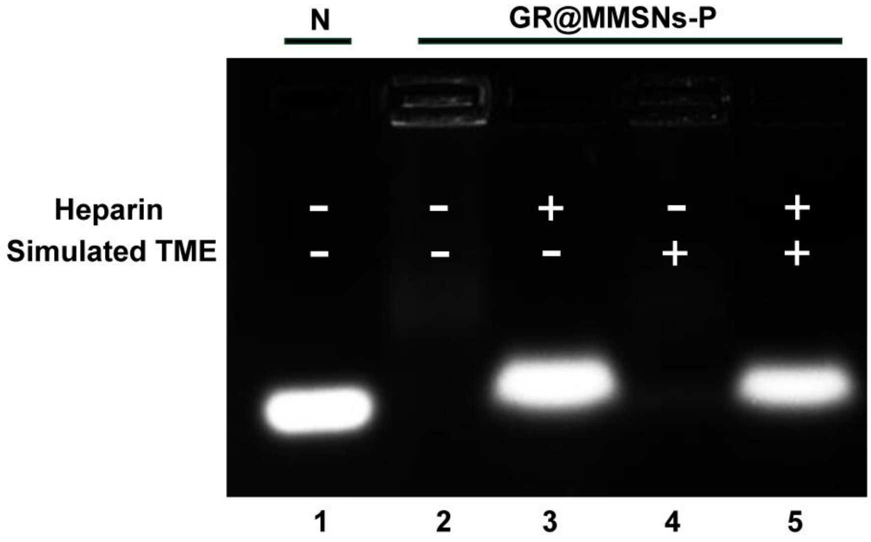

Figure S5. The stability of siRNA in the simulated TME determined by gel retardation assay. N represented naked siRNA. “+” represented the group treated with simulated TME or heparin. “–” represented the group without simulated TME or heparin treatment.

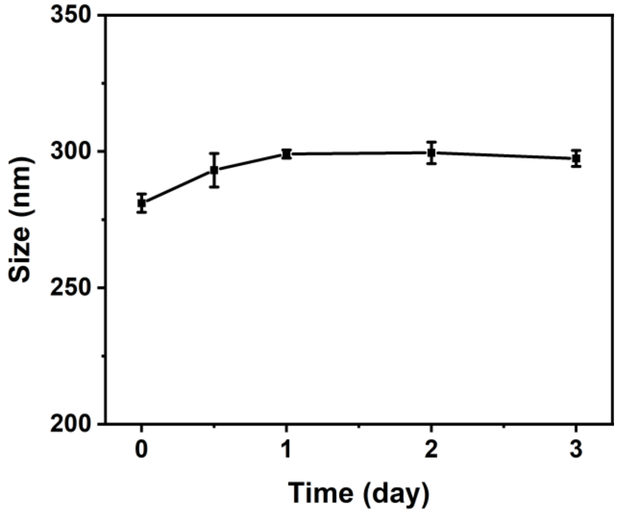

Figure S6. Colloid stability of GR@MMSNs-P in DMEM medium with 10% FBS within 3 days.

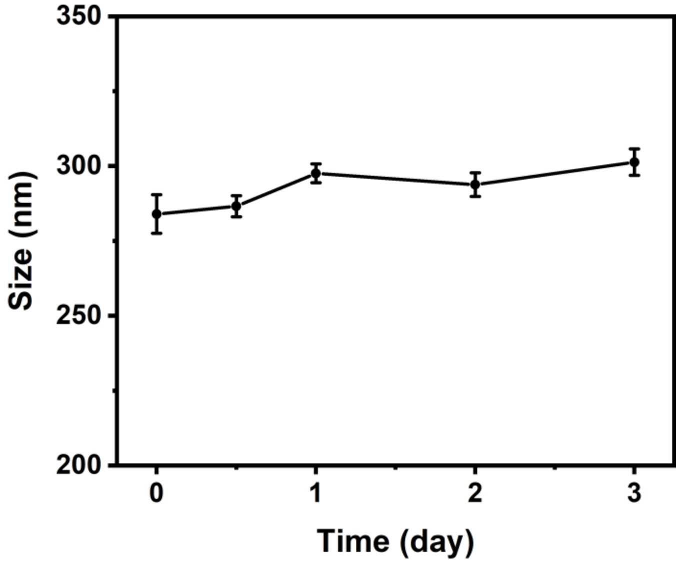

Figure S7. Colloid stability of GR@MMSNs-P in 10% FBS within 3 days.

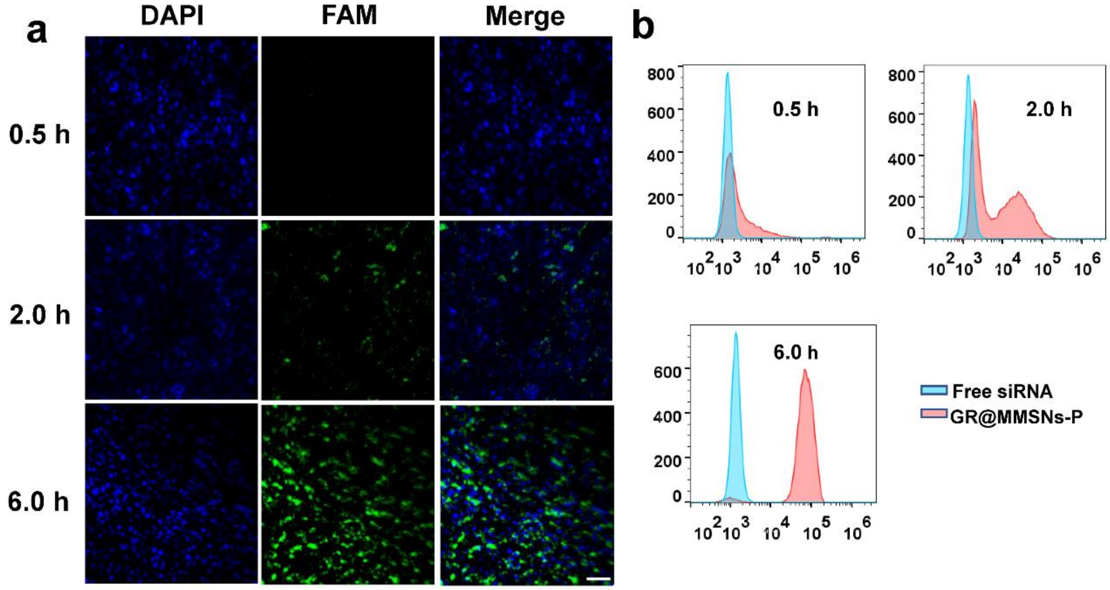

Figure S8. (a) CLSM images of 4T1 cells treated with GR@MMSNs-P for 0.5, 2 and 6 h, respectively. Blue fluorescence: DAPI, green fluorescence: FAM-labelled siRNA. Scale bar, 50 μm. (b) Intracellular uptake amounts of free siRNA and GR@MMSNs-P in 4T1 cells determined by flow cytometry analysis.

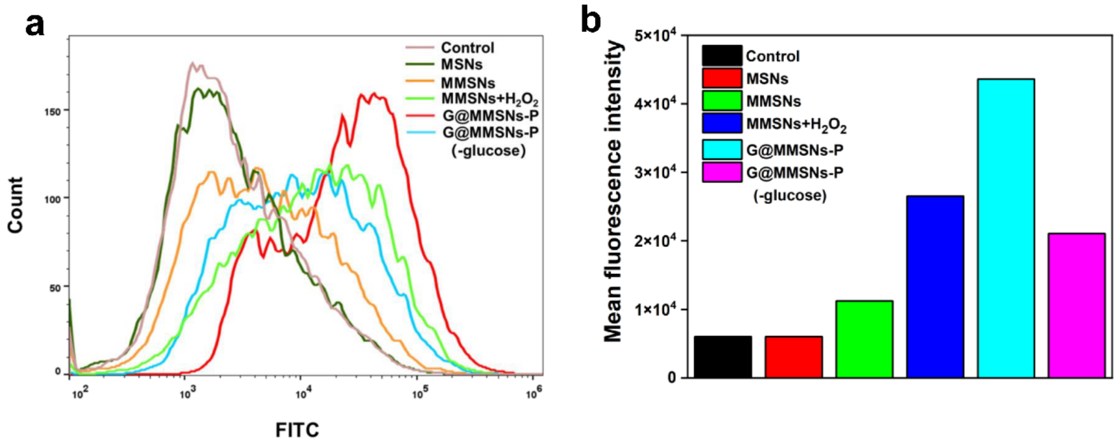

Figure S9. Mean florescence intensity determined by ImageJ software of intracellular ROS variation after different treatments.

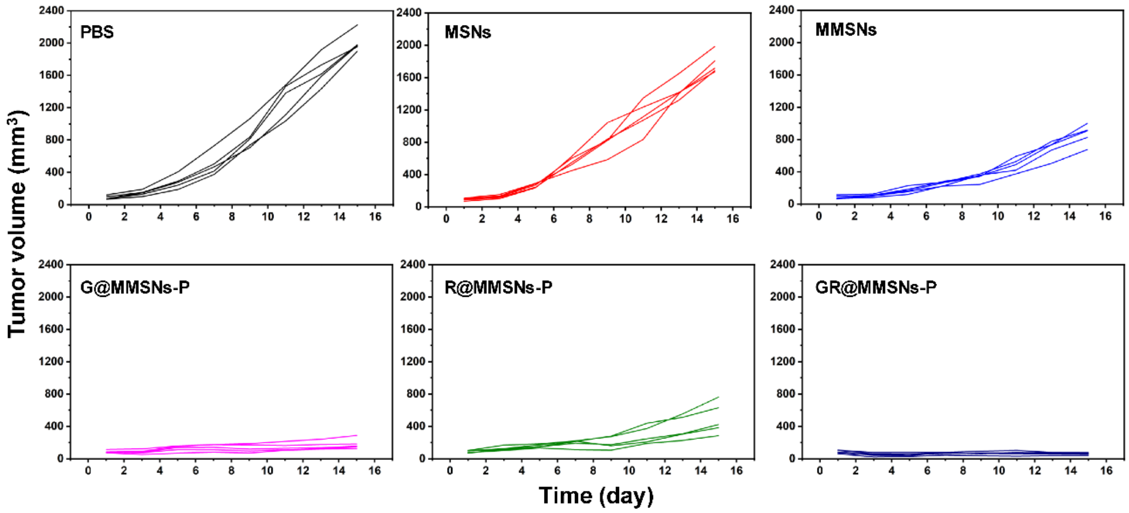

Figure S10. The individual tumor growth kinetics in control and treated groups (n = 5).

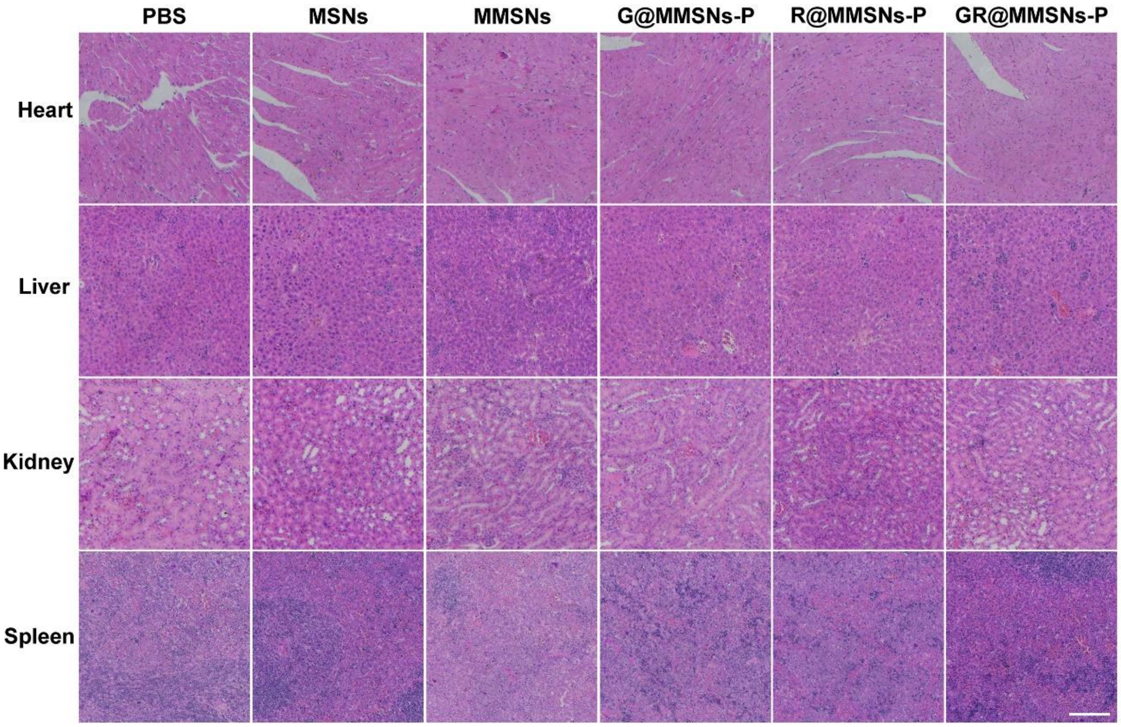

Figure S11. H&E-stained images of major organs (heart, liver, kidney and spleen) collected from the mice at different groups. Scale bar, 100 μm.

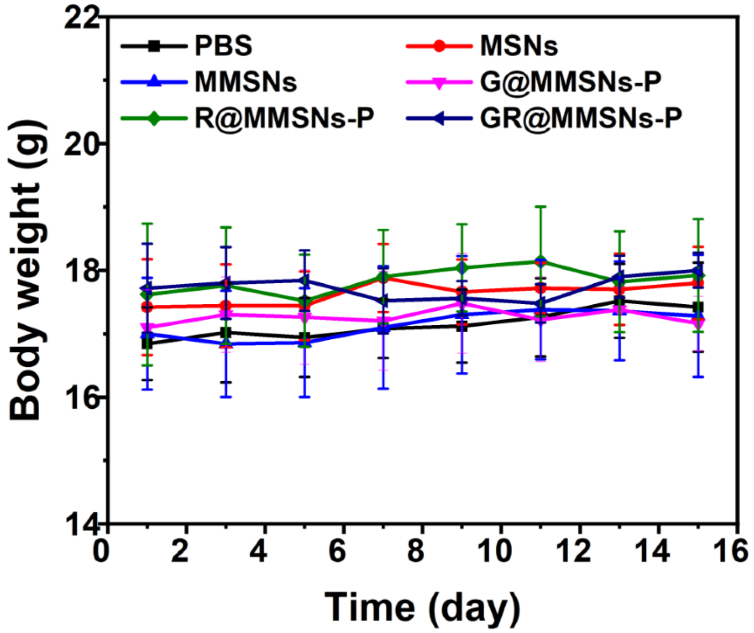

Figure S12. Body weights of the mice in different groups (n = 5).

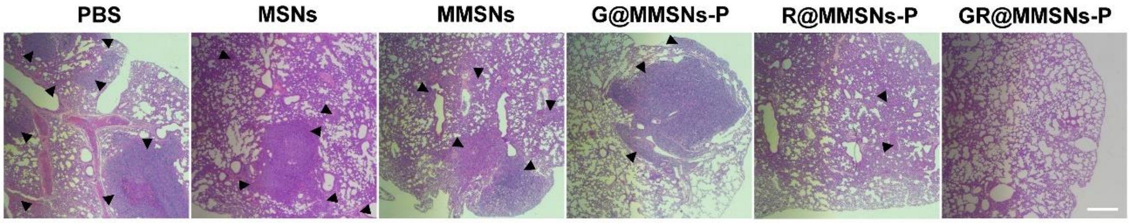

Figure S13. The amplified H&E staining images of lung tissues collected from mice in different groups. Scale bar, 400 μm.
